# Accuracy and Reproducibility of Right Ventricular Quantification in Patients with Pressure and Volume Overload Using Single-Beat Three-Dimensional Echocardiography

**DOI:** 10.1016/j.echo.2014.10.012

**Published:** 2015-03

**Authors:** Daniel S. Knight, Agata E. Grasso, Michael A. Quail, Vivek Muthurangu, Andrew M. Taylor, Christos Toumpanakis, Martyn E. Caplin, J. Gerry Coghlan, Joseph Davar

**Affiliations:** a University College London Medical School, Royal Free Campus, London, United Kingdom; b Department of Cardiology, Royal Free London NHS Foundation Trust, London, United Kingdom; c UCL Centre for Cardiovascular Imaging, University College London, London, United Kingdom; d Neuroendocrine Tumour Unit, Royal Free London NHS Foundation Trust, London, United Kingdom

**Keywords:** Three-dimensional echocardiography, Magnetic resonance imaging, Right ventricular function, Pulmonary hypertension, Carcinoid heart disease, CMRI, Cardiac magnetic resonance imaging, COV, Coefficient of variation, EDV, End-diastolic volume, EF, Ejection fraction, ESV, End-systolic volume, ICC, Intraclass correlation coefficient, PH, Pulmonary hypertension, ROC, Receiver operating characteristic, RV, Right ventricular, RVOT, Right ventricular outflow tract, SV, Stroke volume, TAPSE, Tricuspid annular plane systolic excursion, 3D, Three-dimensional, 3DE, Three-dimensional echocardiography, 2D, Two-dimensional, 2DE, Two-dimensional echocardiography

## Abstract

**Background:**

The right ventricle is a complex structure that is challenging to quantify by two-dimensional (2D) echocardiography. Unlike disk summation three-dimensional (3D) echocardiography (3DE), single-beat 3DE can acquire large volumes at high volume rates in one cardiac cycle, avoiding stitching artifacts or long breath-holds. The aim of this study was to assess the accuracy and test-retest reproducibility of single-beat 3DE for quantifying right ventricular (RV) volumes in adult populations of acquired RV pressure or volume overload, namely, pulmonary hypertension (PH) and carcinoid heart disease, respectively. Three-dimensional and 2D echocardiographic indices were also compared for identifying RV dysfunction in PH.

**Methods:**

A prospective cross-sectional study was performed in 100 individuals who underwent 2D echocardiography, 3DE, and cardiac magnetic resonance imaging: 49 patients with PH, 20 with carcinoid heart disease, 11 with metastatic carcinoid tumors without cardiac involvement, and 20 healthy volunteers. Two operators performed test-retest acquisition and postprocessing for inter- and intraobserver reproducibility in 20 subjects.

**Results:**

RV single-beat 3DE was attainable in 96% of cases, with mean volume rates of 32 to 45 volumes/sec. Bland-Altman analysis of all subjects (presented as mean bias ± 95% limits of agreement) revealed good agreement for end-diastolic volume (−2.3 ± 27.4 mL) and end-systolic volume (5.2 ± 19.0 mL) measured by 3DE and cardiac magnetic resonance imaging, with a tendency to underestimate stroke volume (−7.5 ± 23.6 mL) and ejection fraction (−4.6 ± 13.8%) by 3DE. Subgroup analysis demonstrated a greater bias for volumetric underestimation, particularly in healthy volunteers (end-diastolic volume, −11.9 ± 18.0 mL; stroke volume, −11.2 ± 20.2 mL). Receiver operating characteristic curve analysis showed that 3DE-derived ejection fraction was significantly superior to 2D echocardiographic parameters for identifying RV dysfunction in PH (sensitivity, 94%; specificity, 88%; area under the curve, 0.95; *P* = .031). There was significant interobserver test-retest bias for RV volume underestimation (end-diastolic volume, −12.5 ± 28.1 mL; stroke volume, −10.6 ± 23.2 mL).

**Conclusions:**

Single-beat 3DE is feasible and clinically applicable for volumetric quantification in acquired RV pressure or volume overload. It has improved limits of agreement compared with previous disk summation 3D echocardiographic studies and has incremental value over standard 2D echocardiographic measures for identifying RV dysfunction. Despite the ability to obtain and postprocess a full-volume 3D echocardiographic RV data set, the quality of the raw data did influence the accuracy of the data obtained. The technique performs better with dilated rather than nondilated RV cavities, with a learning curve that might affect the test-retest reproducibility for serial RV studies.

Quantification of right ventricular (RV) size and function is prognostic in congenital and acquired heart disease.^1-4^ The most convenient imaging modality for assessing the right ventricle is two-dimensional (2D) echocardiography (2DE). However, this is limited by the crescentic RV chamber shape and complex geometry, with inflow and outflow portions in different planes.^5,6^ Thus, cardiac magnetic resonance imaging (CMRI) has become the gold-standard imaging modality for RV quantification.^7^ Unfortunately, CMRI is expensive, time consuming, and of limited availability compared with echocardiography.

One possibility to overcome the limitations of 2DE is three-dimensional (3D) echocardiography (3DE), compared against CMRI in a range of congenital and acquired diseases for RV volumetric quantification.^8^ Three-dimensional echocardiography traditionally uses the disk summation method to reconstruct the right ventricle after sequential slice acquisition over consecutive electrocardiographically gated heartbeats.^9^ This technique, however, is limited by breath holding throughout successive cardiac cycles, stitching artifacts during acquisition, and difficulties identifying inlet and outflow regions in the basal slices during postprocessing.^10^

More recently, ultrasound transducer technology allows the real-time acquisition of a 90° × 90° data set in a single cardiac cycle.^11^ We therefore compared RV volumetric quantification by single-beat full-volume 3DE against CMRI in homogenous patient populations of acquired RV pressure and volume overload, namely, pulmonary hypertension (PH) and carcinoid heart disease, respectively. We also sought to determine the potential incremental value of 3DE versus 2DE in PH and to evaluate the test-retest reproducibility of 3DE for both the acquisition and postprocessing components.

## Methods

### Study Population

We performed a prospective cross-sectional study that enrolled 100 participants in sinus rhythm with no contraindications to magnetic resonance imaging, all of whom underwent comprehensive 2DE, single-beat 3DE of the right ventricle, and CMRI within 2 hours of one another. The participants were divided into four subgroups:

• A group of 49 consecutive patients with PH (diagnosed by right heart catheterization as a mean pulmonary artery pressure >25 mm Hg and a pulmonary capillary wedge pressure <15 mm Hg^12^) who presented for diagnosis and/or follow-up of PH by clinical evaluation and/or right heart catheterization as a disease model of RV pressure overload. The etiologies of PH included idiopathic (*n* = 9), connective tissue disease associated (*n* = 32), and chronic thromboembolic disease (*n* = 8). Exclusion criteria comprised clinically significant restrictive or obstructive lung disease identified by pulmonary function tests, arrhythmia, and known independent left-sided cardiac disease unrelated to PH.
• A group of 20 consecutive patients undergoing 2DE for diagnosis and/or follow-up of carcinoid heart disease^13^ were studied as a disease model of RV volume overload.
• A control group of 20 healthy volunteers affiliated with our institution who were age and sex matched to the PH group.
• A control group of 11 age- and sex-matched patients with metastatic neuroendocrine tumor who were screened as negative for carcinoid valvular heart disease.

All control participants were eligible for study inclusion if they had no cardiac symptomatology, had no medical histories of cardiac disease including hypertension, and were not taking any cardiac medications. Normal 2D transthoracic echocardiographic findings were also required to exclude any occult structural cardiac disease before study inclusion.

The institutional research ethics committee approved the study, and informed written consent was obtained from all patients and control subjects.

### 2DE

All patients underwent comprehensive 2D and Doppler transthoracic echocardiography in the left lateral decubitus position using the Acuson Siemens SC2000 cardiac ultrasound system (Siemens Healthcare, Erlangen, Germany), with a 4V1c transducer (frequency bandwidth, 1.25–4.5 MHz). A standard study protocol was followed in conjunction with American Society of Echocardiography guidelines for chamber quantification^14,15^ and the British Society of Echocardiography guidelines for PH assessment^16^ as appropriate. RV function was assessed using M-mode tricuspid annular plane systolic excursion (TAPSE); RV fractional area change, calculated as [(end-diastolic area) − (end-systolic area)/end-diastolic area] × 100; and mean RV free wall peak systolic strain using syngo Vector Velocity Imaging (Siemens Medical Solutions USA, Inc, Mountain View, CA). A three-beat 2D echocardiographic digital clip of an apical four-chamber view optimized for RV visualization was acquired and exported to Velocity Vector Imaging, and 10 to 15 endocardial points were plotted in end-systole from the lateral to the medial tricuspid annulus.^17^ The adequacy of speckle-tracking was visually checked and manually adjusted as required.

### 3DE

#### Image Acquisition

Single-beat full-volume 3D echocardiographic RV data sets were acquired using the 4Z1c matrix-array transducer (frequency bandwidth, 1.5–3.5 MHz; maximum depth, 30 cm; maximum field of view, 90° × 90°). Probe position started from the apical four-chamber view with the patient in the left lateral decubitus position. Both the patient and transducer positions were subsequently modified for optimal simultaneous visualization of the tricuspid valve, cardiac apex, infundibulum, and RV outflow tract (RVOT) as assessed by the real-time 2D four-chamber, basal sagittal, and coronal views, and by inclusion of the RV chamber in the pyramidal data set. In our experience, a more lateral apical window with posterior tilt of the probe tail was beneficial to visualize the infundibulum and RVOT in the coronal window. Image depth and sector width were adjusted for maximal visualization of the right ventricle at the highest volume rate. At least three 3D echocardiographic RV data sets were acquired during a breath-hold to ensure optimal image quality, which was subjectively graded on a five-point scale ranging from zero (very poor) to four (perfect).^18^ A score of two or less was attributed if ultrasound dropout was evident in greater than half of the RVOT border.

#### Postprocessing

**Figure 1:**
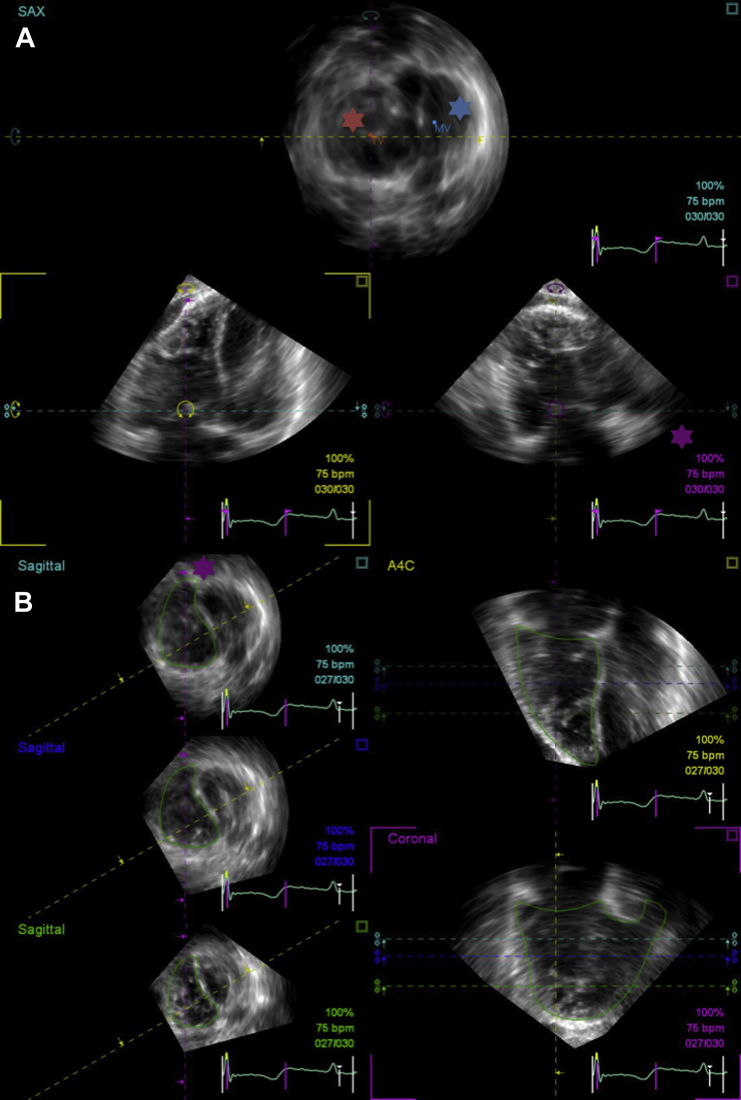
Example of the stepwise process of RV reconstruction by 3DE. **(A)** The cavity area is optimized in the three orthogonal views, and landmarks are identified (mitral valve indicated by the *blue asterisk*, tricuspid valve indicated by the *red asterisk*). Note the RVOT seen in the coronal view (indicated by the *purple asterisk*). **(B)** Having traced endocardial borders in three orthogonal views, the semiautomated border tracking results are displayed for inspection. Note how the purple guideline (indicated by the *purple asterisk*) bisects the tricuspid valve and RVOT in the short-axis views. This corresponds to the coronal RV reconstruction (*highlighted*), with clearly delineated RV inflow and outflow portions. *A4C*, Apical four-chamber.

Full-volume 3D echocardiographic RV data sets were imported into the on-cart RV Analysis application. Manual adjustment of the RV data set was initially required to ensure the correct orientation of four-chamber, sagittal, and coronal slices; maximize the RV cavity area and identify the most apical RV view on visual assessment of the four-chamber window; and allow the identification of cardiac landmarks. This process was performed in a stepwise approach by rotation and angulation of the four-chamber window, with manipulation of this plane causing the simultaneous adjustment of the other two (sagittal and coronal) orthogonal planes (Figure 1A). Both atrioventricular valves followed by the left ventricular apex were identified as anatomic landmarks. When the apex of a dilated right ventricle overrode that of the left ventricle, the most apical cardiac point was identified with the left ventricular apex marker. End-diastolic and end-systolic frames were assigned by visual identification of the largest and smallest RV four-chamber areas, respectively.

Endocardial RV borders were traced at end-diastole and end-systole in four-chamber, basal sagittal, and coronal views. The software algorithm obliges the operator to intersect the endocardial border tracing in sequential views with crosshair reference markers that are positioned in response to endocardial border traces from a preceding view. Therefore, correction of a previous slice tracing was undertaken when a crosshair position suggested a prior tracing error. Trabeculae were included in the blood pool volume. To assist with RVOT delineation in the basal sagittal view, the insertion point of the RV myocardium at the interventricular septum was routinely included in the endocardial tracing.

At the final stage, the algorithm presents the results of semiautomated contour tracking for the four-chamber, coronal and basal, middle and apical short-axis views. Misalignment of endocardial contours prompted identification of the region of suboptimal tracking followed by manual correction of the original tracing. Automated volumetric reconstruction was accepted only once the semiautomated endocardial border tracking was visually satisfactory and represented meaningful RV shapes in all views (Figure 1B), as optimization of this final reconstruction stage significantly affects the results generated.^19^ The algorithm from which the final RV volume is generated has been previously described.^20,21^

#### Test-Retest Reproducibility of 3DE

Reproducibility was studied in 20 randomly selected subjects (14 with PH, one with carcinoid heart disease, and five healthy volunteers) for both the 3D echocardiographic acquisition and postprocessing stages by two independent sonographers (D.S.K. and A.E.G.), as described previously.^22^ The two sonographers had equal experience with 2DE but differing levels of experience with 3D echocardiographic RV full-volume acquisition (10 and 3 months, respectively). Sonographer 1 (D.S.K.) obtained a 3D echocardiographic RV data set, after which sonographer 2 (A.E.G.) independently obtained a 3D echocardiographic RV data set. Then, sonographer 1 acquired a second separate 3D echocardiographic RV data set. The sonographers, who were blinded to each other’s results, performed postprocessing of their own 3D echocardiographic RV data sets. Data sets for intraobserver test-retest reproducibility were postprocessed separately at time intervals of >2 weeks.

### Cardiac MRI

#### Image Acquisition

All cardiac magnetic resonance images were acquired using a 1.5-T magnetic resonance scanner (Avanto; Siemens Healthcare) using a 12-element phased-array coil for signal reception and the body coil for signal transmission. A vector electrocardiographic system was used for cardiac gating. Ventricular volumes and great vessel flow were measured in all patients. Volumetric RV data were obtained using either retrospectively gated balanced steady-state free precession (*n* = 19) cine imaging of contiguous short-axis slices^23^ or real-time radial *k*-*t* sensitivity encoding imaging (*n* = 81) of contiguous transaxial slices^24^ depending on the pathology under investigation and the patient’s ability to hold his or her breath. Real-time radial *k*-*t* sensitivity imaging allows the collection of high spatiotemporal resolution real-time images during free breathing and is part of the standard clinical CMRI work flow at our institution in the pediatric PH population.^25^ Transaxial RV slices were preferred for the PH cohort and their respective control population because of the relative preservation of longitudinal versus radial RV function that is manifest in this condition.^26^ Blood flow data were acquired in the ascending aorta, in the right and left branch pulmonary arteries, and at the level of the atrioventricular valves using a velocity-encoded prospectively triggered spiral phase-contrast magnetic resonance flow sequence.^27^ This provided an internal check for the RV volumetric data.

#### Postprocessing

All image postprocessing was performed using “in-house” plug-ins for the open-source OsiriX Digital Imaging and Communications in Medicine software.^24,28,29^ Endocardial RV borders were traced manually at end-diastole and end-systole, the time points of which were identified by the largest and smallest RV cavity areas, respectively. The inclusion of RV trabeculae was the same as that performed by 3D echocardiographic postprocessing. Ventricular stroke volume (SV) was the difference between end-diastolic volume (EDV) and end-systolic volume (ESV), and ejection fraction (EF) was calculated as (SV/EDV) × 100. Phase-contrast magnetic resonance flow data were segmented using a semiautomatic vessel edge detection algorithm with manual operator correction.^28^ The CMRI data sets for the patients who underwent 3D echocardiographic test-retest reproducibility scans were also tested for interobserver (D.S.K. and M.A.Q.) and intraobserver postprocessing reproducibility.

### Statistical Analysis

Statistical analysis was performed using SPSS version 21.0 (IBM Corporation, Armonk, NY) and Prism 6.0b for Mac (GraphPad Software, Inc, La Jolla, CA). Normally distributed continuous data were expressed as mean ± SD. Systematic differences between measurements were evaluated with Student paired *t* tests (two tailed), with Pearson correlation coefficients used to assess the relationship between 3DE- and CMRI-derived RV volumes and EF. Differences between the four participant subgroups were analyzed using one-way analysis of variance, with the Tukey post hoc tests identifying which specific means differed. *P* values < .05 were considered statistically significant. Image scoring data were nonparametrically distributed, represented by medians with 25th and 75th percentiles. Rank sum tests were used for comparisons of image scoring data, with the Mann-Whitney *U* test and the Kruskal-Wallis test used for comparisons of two and three independent groups, respectively.

Intermodality, interobserver, and intraobserver agreement was studied using the Bland-Altman method,^30^ whereby the mean difference was presented as the bias and 95% limits of agreement around the bias expressed as the mean difference ± 1.96 SDs. Differences between test-retest measurements were analyzed by one-way repeated measures analysis of variance, with the Bonferroni post hoc test identifying which specific means differed. The Greenhouse-Geisser correction was used if the assumption of sphericity had been violated. Test-retest variability was expressed using intraclass correlation coefficients (ICC), relative differences, and coefficients of variation (COVs). The ICC was quantified by the two-way random-effects model with absolute agreement. An ICC > 0.85 was considered excellent. Relative differences were calculated by taking the absolute difference between two observations divided by the mean of the repeated observations and expressed as a percentage. COVs were calculated as the standard deviation of the difference between two acquisitions divided by their mean value and expressed as a percentage.^31^ A COV ≤ 10% was considered excellent.

Receiver operating characteristic (ROC) curves were derived for 2D and 3D echocardiographic parameters to identify CMRI-derived RV EFs of <50% in patients with PH and healthy volunteers.^32^ Patients with carcinoid disease were excluded from this analysis to avoid the confounding effects of severe valvular regurgitation on ventricular function. The area under the ROC curve for an echocardiographic parameter is presented together with the optimal cutoff threshold for detecting CMRI-derived RV EF < 50%, defined as the value of the parameter that corresponded to the highest sum of sensitivity and specificity. The Delong method was used to compare the areas under the curve between ROC curves^33^ (Analyse-it Software, Ltd, Leeds, United Kingdom).

## Results

### Population Characteristics and 3DE Technical Data

**Figure 2:**
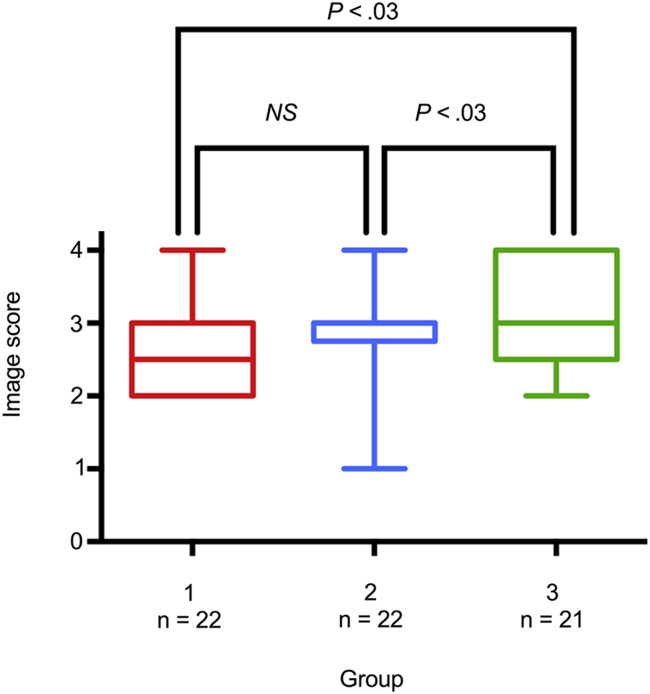
*Box*-and-*whisker* plots of subjective image quality scores among three successive subgroups of patients (group 1 acquired in the earliest phase of the study, group 3 in the latest phase of the study). Image quality significantly improved with increasing experience with 3DE (*P* = .031).

**Figure 3:**
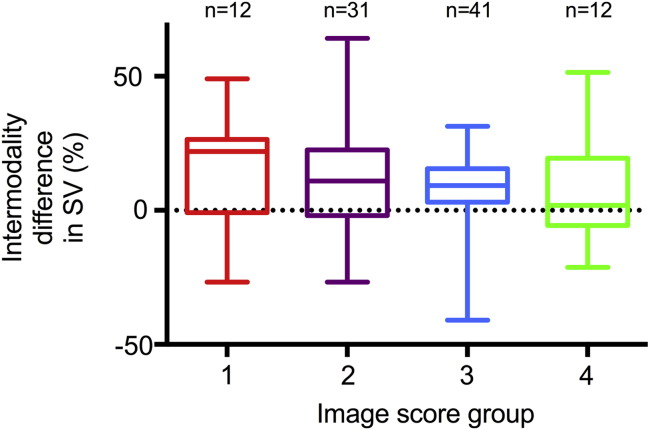
*Box*-and-*whisker* plots of differences in stroke volume between modalities (expressed as a percentage of the CMRI reference value) for image scoring groups 1 to 4. There was a trend, albeit not statistically significant, for the intermodality difference to increase with reductions in subjective image score. Median percentage intermodality differences in stroke volume by image score group were as follows: group 1, 22% (interquartile range [IQR], −1% to 26%); group 2, 11% (IQR, −2% to 23%); group 3, 9% (IQR, 3% to 16%); group 4, 2% (IQR, −6% to 19%).

Of 100 individuals who were recruited, four had unobtainable RV echocardiographic windows. The clinical characteristics and 3D echocardiographic technical data of the final cohort of 96 subjects are presented in Table 1. Patients with PH had significantly larger and impaired right ventricles than controls, whereas the right ventricles of patients with carcinoid heart disease were also significantly dilated but with preserved EFs. The dilated right ventricles of the patient groups resulted in a significantly lower mean volume rate compared with controls because of the greater 3D sector angles (*P* < .001), but the median image quality score was significantly higher among patients (3.00; interquartile range, 2.00–3.00) than controls (2.00 interquartile range, 1.00–3.00) (*P* < .001). The image quality among three successive, equally populated subgroups of patients significantly improved with increasing experience with 3DE (Figure 2; *P* = .031). There was a trend, albeit not statistically significant, for greater differences in SV between modalities with worse subjective image scores (Figure 3; *P* < .13 for percentage intermodality difference in SV for image score groups 1 and 2 combined vs groups 3 and 4 combined).

### Volumetric Analysis by 3DE versus CMRI

**Figure 4:**
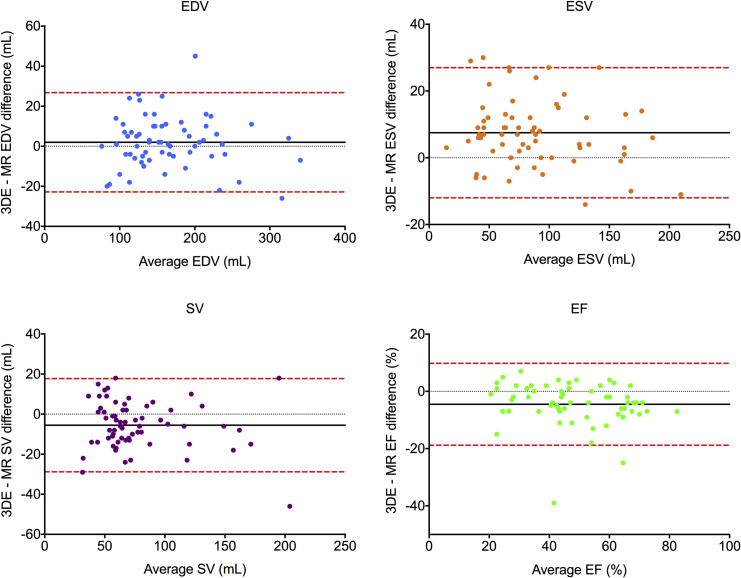
Bland-Altman analysis of bias (*black solid line*) and 95% limits of agreement (*red dashed line*) for 3DE versus CMRI quantification of RV EDV, ESV, SV, and EF in patients with PH and carcinoid heart disease.

**Figure 5:**
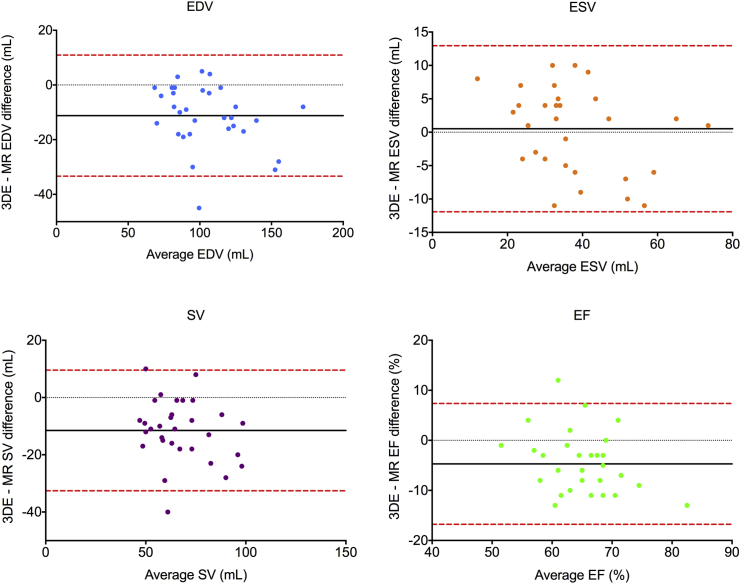
Bland-Altman analysis of bias (*black solid line*) and 95% limits of agreement (*red dashed line*) for 3DE versus CMRI quantification of RV EDV, ESV, SV, and EF for subjects in the control populations.

Correlation coefficients showed good to excellent correlations between modalities for RV metrics in patient groups and moderate to good correlations for control subjects (Table 2). RV volumes and EFs by 3DE showed differences with CMRI in both patient groups, with a bias for underestimating SV and EF but with overall acceptable limits of agreement (Figure 4). By contrast, 3DE underestimated EDV for control subjects (Table 3), with a consequent negative bias for quantifying SV in this group (Figure 5).

### RV Quantification by 3DE and 2DE versus CMRI

**Figure 6:**
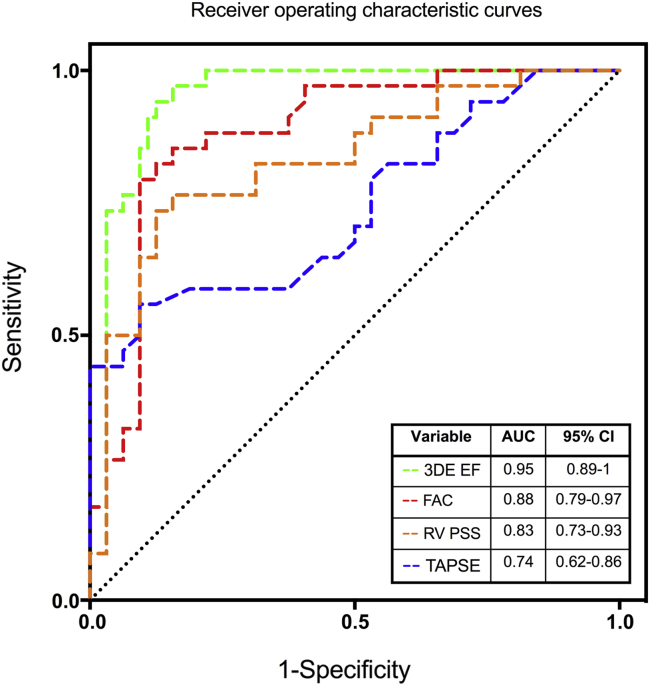
ROC curves for 3DE, fractional area change (FAC), RV free wall peak systolic strain by speckle-tracking echocardiography, and TAPSE to identify RV dysfunction (defined as RV EF < 50% on CMRI).

Three-dimensional echocardiographic EF was the most superior echocardiographic parameter for identifying CMRI-derived RV EF < 50% (Figure 6; *P* = .031), with sensitivity of 94%. A fractional area change of 39% (sensitivity, 85%) was the best conventional 2D echocardiographic measure, superior to both peak systolic strain and TAPSE (*P* = .0443). TAPSE was the weakest marker to predict CMRI-derived RV EF < 50%, with sensitivity of 56% at a cutoff threshold of 19 mm.

### Test-Retest Intraobserver and Interobserver Reproducibility

Limits of agreement were acceptable for intra- and interobserver 3D echocardiographic studies, with good to excellent ICCs (Table 4). However, there was a significant interobserver bias for underestimating RV EDV (*P* = .001; Table 5) that resulted in underestimation of SV (*P* = .002) and EF (*P* = .033), with accompanying large interobserver COVs and relative differences. Moreover, despite no significant differences between intraobserver EDV and ESV, the differences translated into statistically significant test-retest differences for SV (*P* = .032) and EF (*P* = .005). The interobserver and intraobserver reproducibility for RV volumes and EF by CMRI showed no significant bias and superior limits of agreement compared with 3DE.

## Discussion

This study demonstrates the feasibility of single-beat full-volume 3DE for RV quantification in, to our knowledge, the largest homogenous acquired RV pressure- and volume-overloaded patient populations. Single-beat 3DE is an agreeable technique compared with CMRI, albeit with significant differences especially in subjects with nondilated right ventricles. Furthermore, 3D echocardiographic parameters are of incremental benefit for RV functional quantification compared with traditional 2DE measures.

Accurate quantitation of RV size and function is important in many congenital and acquired cardiac diseases and is of particular relevance in our study populations. RV size and function are of greater prognostic significance in PH than the afterload to which the right heart is exposed,^34,35^ with RV EF being the key determinant of outcome regardless of changes in pulmonary vascular resistance afforded by pulmonary vasodilator therapy.^36^ Similarly, right heart dilatation is independently associated with poor outcomes in patients with advanced carcinoid heart disease.^37^ However, the right ventricle responds differently to pressure- and volume-overload conditions, with dilatation occurring in both but with relative preservation of function in elevated preload rather than afterload. What remains unclear is to what extent this preserved EF represents normality of function in the presence of severe tricuspid regurgitation, a valvular lesion common to all patients in our carcinoid heart disease cohort.

The incremental benefit of 3DE over 2DE has previously been shown in congenital heart disease,^38^ and single-beat 3DE showed similar added value over 2DE metrics in acquired RV pressure overload. Although this is due in part to equivalent parameters being assessed by 3DE and CMRI, it is, importantly, also a reflection of the limitations of conventional 2D echocardiographic measures. TAPSE had the poorest sensitivity for detecting low RV EF in PH, with a cutoff of 19 mm having the highest combined sensitivity and specificity. This is higher than the recommended threshold of 16 mm for detecting RV dysfunction,^14^ suggesting that TAPSE would have performed worse by current guidelines in our cohort. The rocking motion of the right ventricle in pressure overload can give rise to apparently normal TAPSE values,^39^ and TAPSE also does not account for the radial component of RV function that contributes significantly to RV EF.^26^ By contrast, fractional area change was the most superior 2D echocardiographic marker for identifying RV dysfunction in PH, most likely a reflection of being the only 2D echocardiographic marker that accounts for radial function. These findings are consistent with previous studies comparing 2D echocardiographic markers of RV function in PH^40^ and suggest that 3DE may have an important additive role in assessing RV function.

RV quantification by echocardiography is advantageous through being more readily available and less expensive than CMRI. Since the first use of 3DE for RV volumetric quantification,^18^ improvements in matrix-array transducer technology permit the simultaneous visualization of orthogonal 2D RV planes at the time of acquisition. The technique used in this study allows a pyramidal data set of up to 90° × 90° to be acquired at higher temporal resolutions than previously reported for 3DE.^41^ Acquisition of a full volume in a single heartbeat avoids stitching artifacts associated with acquiring slices over serial heartbeats and also confers the advantage of shorter breath-hold durations. These reasons might explain the narrower limits of agreement for RV volumetric parameters between single-beat 3DE and CMRI compared with previous data from adult PH groups using the disk summation method.^41,42^

The disadvantages of echocardiography include constraints that afford inadequate transthoracic windows, including body habitus, hyperinflated lungs, and chest deformities. Acquisition and postprocessing was feasible in 96% of subjects, consistent with previously reported studies using the technique.^43,44^ However, patients with significant lung disease were excluded to ensure that PH was the predominant disease process in the RV pressure-overload group, and this may in turn have biased the echogenicity of the study population. Although all postprocessed 3D echocardiographic data sets had a reconstructed RV polygon that tracked throughout the cardiac cycle, 45% of studies were judged by subjective image scoring to have some endocardial dropout of the outflow portion of the right ventricle. This was reflected by a trend toward increasing differences in SVs between modalities with decreasing image quality, with a median difference of ≥11% when the RVOT was incompletely visualized. This is a consistent problem with 3DE that has been well documented previously and is due to the anterior position of the right ventricle in the thorax. Postprocessing software extrapolates the endocardial borders during semiautomated border tracking,^45^ and hence although it is possible to analyze data sets with incomplete RVOT visualization, the accuracy of reconstructions will most likely deteriorate with progressive dropout in the outflow tract.

When comparing studies of RV quantification by 3DE, the homogeneity of the study population must be taken into account. Our populations of acquired RV disease were favorable for the 3D echocardiographic postprocessing software algorithm, because it is set up for an adult-shaped right ventricle rather than a congenital heart.^46^ This may be a reason why our limits of agreement were narrower than reported in congenital heart disease.^45^ No substantial bias was observed in either the PH or carcinoid heart disease group, but subgroup analysis showed that EDVs, and consequently SVs, were underestimated in controls. This is despite the higher temporal resolution of images in this group and is likely a result of low spatial resolution with single-beat 3DE. Lower spatial resolution confers less ability to resolve myocardium and trabeculae, thus directing the operator to trace the endocardium further inside the RV cavity and hence underestimate volumes. This is supported by previous data showing greater variability and negative bias for 3DE to quantify RV volumes in nondilated right hearts.^47^ Conversely, RV endocardial delineation is known to be easier in the setting of RV hypertrophy or dilatation for both magnetic resonance imaging and 3DE^41,47^ and is reflected by the higher image quality scores observed with our disease cohorts.

The progressive increase in 3D echocardiographic image quality over the study duration reflects a significant learning curve with the technique, as also described in previous studies.^38^ This is important clinically, as follow-up studies will vary depending on operator experience for both acquisition and postprocessing. Few studies so far have addressed 3D echocardiographic test-retest reproducibility for both the acquisition and postprocessing stages.^22,41^ Our interobserver test-retest study demonstrated a second operator bias for EDV underestimation, conferring lower SV and EF measurements. This was a systematic error likely reflecting relative operator inexperience with the technique. The susceptibility of 3DE to underestimate RV volumes has been well documented,^8^ and our data suggest that operator experience is related to this underestimation.

Furthermore, nonsignificant differences in intraobserver EDV and ESV nevertheless resulted in significant differences in SV and EF when the errors in the raw volumes were combined. Given that small changes in endocardial border delineation are known to confer significant changes in 3DE-derived volumetric parameters in the left ventricle,^48^ this is also likely to be a problem with 3DE reconstruction of the right ventricle too. This is clinically important because a change of as little as 10 mL in SV by CMRI is clinically significant in PH,^49^ but a change of this magnitude may be masked by 3DE’s reproducibility error and/or the degradation of accuracy found with poorer quality 3D echocardiographic data sets. For example, the interobserver measurement of RV SV by 3DE showed a significant bias with a standard deviation more than double that of CMRI. Our CMRI reproducibility data show narrow limits of agreement, with no major bias between observers, consistent with previous reproducibility studies of RV quantification by transaxial slices^50,51^ and sensitive enough to detect small changes in RV indices on serial studies.

### Limitations

This study was a single-center study based on acquisitions made by one sonographer with experience using single-beat 3DE for RV volumetric quantification. As demonstrated by our test-retest reproducibility, results cannot be applied across operators with variable experience in 3D echocardiographic RV analysis. Patients with arrhythmias were excluded because of the extra variability introduced by irregular cardiac cycles when comparing modalities. Single-beat acquisition is advantageous over traditional disk summation techniques that are limited by stitching artifacts due to irregular R-R intervals, and this patient group requires further investigation with the technique.

We found a bias to underestimate cavity size, particularly in control subjects, but the study was not designed to compare the accuracy of the technique in patients with large versus small cavity sizes. This question should be addressed in a separate prospectively designed analysis of large versus small right ventricles. The sensitivity and specificity values for 3DE and 2DE to identify CMRI-derived RVEF < 50% were calculated by applying the ROC cutoff values to the same patients used to derive them as described previously,^38^ hence representing a “best case” scenario. A more appropriate method would be to identify cut-off values using ROC analysis in a derivation group, then prospectively evaluate the cutoff values in a separate test group in whom outcomes could be verified independently. Thus the diagnostic performance of the cutoff values found in this study needs to be confirmed independently.

Finally, the study was not designed to provide CMRI test-retest reproducibility similar to the 3D echocardiographic study design for acquisition and postprocessing. However, CMRI does not have the same acquisition window restrictions inherent to transthoracic echocardiography, as contiguous transaxial RV slices of fixed thickness are acquired from the base of the right heart to the main pulmonary artery with the patient in the supine position. However, this difference in technique methodology is a potential source of discrepancy, with the reference standard of CMRI building volumes from multiple slices compared with the full-volume data sets of 3DE.^10^

## Conclusions

Single-beat full-volume 3DE is a feasible technique for quantifying RV size and function in acquired right heart pressure and volume overload. The limits of agreement of 3DE are acceptable compared with CMRI but may not be sensitive enough to detect small yet clinically significant responses to treatment demonstrated by this modality.^49^ The test-retest reproducibility of 3DE suggests a significant learning curve that needs to be considered, and thus results cannot necessarily be extrapolated to less experienced operators. Nevertheless, 3DE showed incremental benefit over conventional 2D echocardiographic measures, suggesting an important role in assessing acquired RV pathology. Future work should focus on improving spatial resolution to optimize RV endocardial delineation, in particular for adequate visualization of the RVOT in nondilated right ventricles.

## Figures and Tables

**Table 1:** Clinical characteristics of study populations

Variable	PH (*n* = 46)	Carcinoid heart disease (*n* = 19)	Healthy volunteers (*n* = 20)	Carcinoid (no valvulopathy) (*n* = 11)	*P*∗
Age (y)	56 ± 13	63 ± 8	50 ± 12	59 ± 10	
Women	35 (76%)	7 (37%)	15 (75%)	7 (64%)	
Height (cm)	164 ± 9	171 ± 10	169 ± 8	168 ± 10	.035
Weight (kg)	69 ± 17	72 ± 18	72 ± 12	77 ± 20	.54
Body surface area (m^2^)	1.8 ± 0.2	1.8 ± 0.3	1.8 ± 0.2	1.9 ± 0.3	.37
Heart rate (beats/min)	74 ± 14	67 ± 13	68 ± 9	69 ± 12	.19
Mean PASP on RHC (mm Hg)	44 ± 16				
Pulmonary vasodilators					
Endothelin antagonist	21 (46%)				
PDE-5 antagonist	31 (67%)				
Prostanoid infusion	2 (4%)				
Oral prostanoid	1 (2%)				
Prostaglandin receptor agonist	1 (2%)				
Carcinoid heart disease: affected valves	TV = 19 (100%), PV = 13 (68%), MV = 3 (16%), AV = 3 (16%)				
EDV (mL/m^2^)	87 ± 26	100 ± 35	64 ± 14	52 ± 8	<.0001
ESV (mL/m^2^)	52 ± 25	33 ± 15	22 ± 7	16 ± 5	<.0001
EF (%)	43 ± 14	68 ± 7	65 ± 7	71 ± 7	<.0001
3D echocardiographic temporal resolution (volumes/sec)	34 ± 5	32 ± 7	40 ± 5	45 ± 6	<.0001

*AV*, Aortic valve; *ESV*, end-systolic volume; *MV*, mitral valve; *PASP*, pulmonary artery systolic pressure; *PDE-5*, phosphodiesterase-5; *PV*, pulmonary valve; *RHC*, right heart catheterization; *TV*, tricuspid valve. Data are expressed as mean ± SD or as number (percentage).

∗ One-way analysis between groups.

**Table 2:** RV volumes and EFs by single-beat full-volume 3DE versus CMRI

Group	Measurement	3DE	CMRI	*P*∗
PH	EDV (mL)	158 ± 53	154 ± 52	.043
	ESV (mL)	100 ± 44	92 ± 47	<.0001
	SV (mL)	58 ± 18	63 ± 17	.011
	EF (%)	39 ± 11	43 ± 14	.00029
Carcinoid heart disease	EDV (mL)	182 ± 69	185 ± 71	.21
	ESV (mL)	67 ± 28	62 ± 3	.01
	SV (mL)	115 ± 42	124 ± 45	.014
	EF (%)	64 ± 5	68 ± 7	.001
Healthy volunteers	EDV (mL)	105 ± 26	117 ± 27	<.0001
	ESV (mL)	41 ± 12	41 ± 14	.80
	SV (mL)	65 ± 16	76 ± 18	<.0001
	EF (%)	61 ± 5	65 ± 7	.014
Carcinoid (no valvulopathy)	EDV (mL)	88 ± 21	98 ± 27	.05
	ESV (mL)	32 ± 13	30 ± 14	.24
	SV (mL)	56 ± 10	68 ± 14	.009
	EF (%)	64 ± 7	71 ± 7	.004

*ESV*, End-systolic volume. Data are expressed as mean ± SD.

∗ Paired Student t tests.

**Table 3:** Bias, limits of agreement, and correlation between single-beat full-volume 3DE and CMRI for RV volumes and EFs

Group	Measurements	Bias ± SD	Limits of agreement	*r*	*P*∗
All subjects	EDV (mL)	−2.3 ± 13.7	−29.1 to 24.5	0.97	<.0001
	ESV (mL)	5.2 ± 9.5	−13.4 to 23.9	0.98	<.0001
	SV (mL)	−7.5 ± 11.8	−30.6 to 15.7	0.94	<.0001
	EF (%)	−4.6 ± 6.9	−18.2 to 9.0	0.91	<.0001
PH	EDV (mL)	4.0 ± 13.1	−21.6 to 29.7	0.97	<.0001
	ESV (mL)	8.4 ± 10.6	−12. 3 to 29.1	0.98	<.0001
	SV (mL)	−4.3 ± 10.8	−25.5 to 17.0	0.82	<.0001
	EF (%)	−4.8 ± 8.3	−21.1 to 11.5	0.81	<.0001
Carcinoid heart disease	EDV (mL)	−3.1 ± 10.1	−22.9 to 16.8	0.99	<.0001
	ESV (mL)	5.4 ± 8.2	−10.6 to 21.4	0.96	<.0001
	SV (mL)	−8.6 ± 13.9	−35.9 to 18.6	0.95	<.0001
	EF (%)	−3.8 ± 4.1	−11.9 to 4.2	0.82	<.0001
Healthy volunteers	EDV (mL)	−11.9 ± 9.0	−29.5 to 5.8	0.94	<.0001
	ESV (mL)	−0.4 ± 6.7	−13.6 to 12.9	0.88	<.0001
	SV (mL)	−11.2 ± 10.1	−31.0 to 8.7	0.84	<.0001
	EF (%)	−3.9 ± 6.5	−16.6 to 8.8	0.51	.021
Carcinoid (no valvulopathy)	EDV (mL)	−10.1 ± 15.0	−39.6 to 19.4	0.84	.001
	ESV (mL)	2.1 ± 5.5	−8.7 to 12.9	0.92	<.0001
	SV (mL)	−12.2 ± 12.3	−36.3 to 11.9	0.53	.096
	EF (%)	−6.2 ± 5.6	−17.1 to 4.7	0.69	.019

*ESV*, End-systolic volume.

∗ Pearson correlation coefficient.

**Table 4:** Interobserver and intraobserver reproducibility for RV volumes and EF by 3DE and CMRI

Variable	EDV (mL)	ESV (mL)	SV (mL)	EF (%)
3DE intraobserver				
ICC	0.992	0.974	0.96	0.906
COV (%)	3.0	6.6	8.0	6.9
RD (%)	4.3	9.4	11.3	9.8
Bias	−0.2	4.6	−4.7	−3.6
LOA	−16.2 to 15.8	−12.8 to 22.0	−19.0 to 9.7	−12.2 to 5.0
SD	8.2	8.9	7.3	4.4
CMRI intraobserver				
Bias	−2.6	−2.4	−0.1	0.7
LOA	−15.4 to 10.2	−11.3 to 6.5	−11.8 to 11.6	−5.9 to 7.2
SD	6.5	4.6	6.0	3.4
3DE interobserver				
ICC	0.955	0.965	0.867	0.827
COV (%)	7.7	8.0	16.6	9.4
RD (%)	10.3	11.4	23.5	13.3
Bias	−12.5	−2.0	−10.6	−4.0
LOA	−40.0 to 15.1	−24.0 to 20.1	−33.2 to 12.1	−16.2 to 8.3
SD	14.1	11.3	11.6	6.3
CMRI interobserver				
Bias	−1.9	−2.80	1.1	0.9
LOA	−18.2 to 14.4	−13.1 to 7.5	−9.3 to 11.5	−4.1 to 5.8
SD	8.3	5.2	5.3	2.5

*ESV*, End-systolic volume; *LOA*, limits of agreement; *RD*, relative difference.

**Table 5:** Interobserver and intraobserver test-retest RV metrics by 3DE

	Sonographer 1			Sonographer 2		
Variable	First (S1.1)	Second (S1.2)	*P*∗ S1.1 vs S1.2	Acquisition	*P*∗ vs S1.1	*P*∗ vs S1.2
EDV (mL)	145 ± 63	145 ± 62	NS	133 ± 59	.003	.003
ESV (mL)	78 ± 44	83 ± 42	NS	76 ± 39	NS	NS
SV (mL)	67 ± 31	63 ± 29	.032	57 ± 27	.002	.046
EF (%)	48 ± 13	44 ± 12	.005	44 ± 11	.033	NS

*ESV*, End-systolic volume. Data are expressed as mean ± SD.

∗ One-way repeated-measures analysis of variance with Bonferroni post hoc test.
